# Targeting Macrophages in Immunotherapy: The Ascent of CAR-Macrophages

**DOI:** 10.3390/ijms27031292

**Published:** 2026-01-28

**Authors:** Vinod Nadella, Anu Sharma

**Affiliations:** 1 Department of Host Microbe Interactions, St. Jude Children’s Research Hospital, Memphis, TN 38103, USA; 2 Department of Oncology, St. Jude Children’s Research Hospital, Memphis, TN 38103, USA

**Keywords:** cancer, macrophages, immunotherapy, chimeric antigen receptor, tumor microenvironment, cell therapy engineering, solid tumors

## Abstract

Chimeric antigen receptor (CAR)-engineered immune cell therapies have revolutionized cancer treatment, with CAR-T cells demonstrating remarkable efficacy against hematological malignancies. However, the effectiveness of CAR-T and other lymphocyte-based therapies against solid tumors remains limited, primarily due to the immunosuppressive tumor microenvironment and poor infiltration of effector cells. Recently, CAR-macrophage (CAR-M) immunotherapy has emerged as a promising strategy to overcome these barriers. Leveraging the innate tumor-homing ability, phagocytic function, and antigen-presenting capacity of macrophages, CAR-M therapies offer unique advantages for targeting solid tumors. This review provides a comprehensive overview of the development and current state of CAR-Macrophage immunotherapy, including advances in CAR design and macrophage engineering, preclinical and clinical progress, and mechanistic insights into their anti-tumor activity. The review critically examined both the benefits and limitations of CAR-M approaches, addressing persistent challenges such as cell sourcing, durability, and safety, while also exploring innovative strategies to enhance therapeutic efficacy. Finally, future perspectives and the potential clinical impact of CAR-macrophage therapies were outlined, underscoring their emerging role in the evolving landscape of cancer immunotherapy.

## 1. Introduction

Immunotherapy has revolutionized cancer treatment, bringing renewed hope to patients with both hematological and solid malignancies. Among several advances, chimeric antigen receptor (CAR) T-cell therapy has achieved remarkable success, particularly in B-cell malignancies [1,2,3,4]. By redirecting T-cell specificity to recognize and eliminate cancer cells, CAR-T therapy has produced unprecedented remission rates in cases previously considered refractory [5,6]. To date, the US FDA has approved six commercial CAR-T cell products for a range of relapsed or refractory cancers [5,7,8]. Nevertheless, the application of CAR-T and other CAR-engineered lymphocyte therapies to solid tumors remains challenging, largely due to limited trafficking and infiltration into tumor sites, antigen heterogeneity, and the profoundly immunosuppressive tumor microenvironment (TME). These barriers underscore the need for alternative approaches to effectively target solid malignancies.

Given these limitations, attention has shifted to macrophages, which serve as vital sentinels and coordinators of the immune system. Renowned for their ability to identify, engulf, and eliminate pathogenic microbes as well as dead or damaged cells, macrophages play a crucial role in maintaining tissue integrity. Their inherent capacity to infiltrate tissues, modulate the local immune environment, and orchestrate both innate and adaptive immune responses positions them as promising candidates for next-generation cellular immunotherapies targeting solid tumors. Macrophage activation is initiated by a range of signals, including microbial components known as pathogen-associated molecular patterns (PAMPs), cytokines such as interferon-γ primarily released by helper T cells, signals from natural killer and dendritic cells, and damage-associated molecular patterns (DAMPs) from injured or apoptotic cells. Once activated, macrophages display robust phagocytic activity, internalizing pathogens and debris into phagolysosomes where they are degraded by reactive oxygen species, nitric oxide, and proteolytic enzymes [9]. Beyond pathogen clearance, macrophages are pivotal in antigen presentation, processing microbial antigens and displaying them on MHC molecules to both CD4^+^ and CD8^+^ T cells, effectively bridging innate and adaptive immunity. Additionally, activated macrophages secrete a repertoire of pro-inflammatory cytokines (such as IL-1, IL-6, and TNF-α) and chemokines that recruit and activate other immune cells, while upregulating membrane-bound molecules including MHC, costimulatory proteins (CD80, CD86), and Fc/complement receptors to enhance immune interactions. Through these multifaceted roles, macrophages not only eradicate pathogens directly but also coordinate the broader immune response, ensuring effective pathogen elimination and the activation of adaptive immunity [10]. Moreover, the synergistic role of macrophages in facilitating effective antitumor CAR-T cell responses is well documented. Notably, studies have shown that complete depletion of brain macrophages using a CSF1R inhibitor abrogates the antitumor activity of CAR T cells [11]. These findings underscore the indispensable contribution of macrophages to the overall efficacy of CAR-based immunotherapies and highlight the importance of harnessing their functions to achieve optimal therapeutic outcomes.

Engineering of macrophages with CARs creating CAR-M has emerged as an innovative approach to cancer immunotherapy [12]. CAR-M combine the innate abilities of macrophages to infiltrate solid tumors, phagocytose cancer cells, and modulate the immune microenvironment, with the antigen specificity conferred by CAR technology. Early studies suggest that CAR-M therapies may overcome some of the key limitations faced by CAR-T and CAR-NK therapies, particularly in the context of solid tumors [13,14,15]. Despite the promise of this approach, CAR-M therapy is still in its infancy, with many scientific, technical, and clinical challenges to address. These include optimizing methods for genetic modification, ensuring persistence and function of engineered macrophages, and minimizing potential toxicity or off-target effects. Furthermore, scalable manufacturing and regulatory considerations remain areas of active investigation.

This review addresses a comprehensive overview of the current landscape of CAR-macrophage immunotherapy, exploring the biological rationale for targeting macrophages, recent progress in CAR design and engineering, and findings from both preclinical and clinical studies. It also addresses key challenges in the field while discusses emerging solutions, ultimately highlighting the future directions for research and clinical application in this rapidly evolving area of cancer therapy.

## 2. Biology of Macrophages in Cancer

Macrophages are versatile innate immune cells that play essential roles in tissue homeostasis, defense against pathogens, and the orchestration of immune responses [16,17,18]. In the context of cancer, macrophages are among the most abundant immune cells within the TME, where they are referred to as TAMs [19]. Macrophages within tumors can arise from two primary sources, tissue-resident macrophages originating from embryonic precursors, and monocyte-derived macrophages recruited from the circulation [20,21]. Tumor cells and stromal components secrete chemokines such as CCL2, CSF-1, and VEGF, which attract monocytes to the tumor site, where they subsequently differentiate into TAMs under the influence of the local microenvironment [22,23,24]. The remarkable functional plasticity of macrophages and their responsiveness to diverse environmental clues make them key regulators of tumor progression and therapeutic outcomes.

Macrophages display remarkable phenotypic and functional heterogeneity, which is often described along a continuum between two extremes: classically activated, proinflammatory M1 macrophages and alternatively activated, anti-inflammatory M2 macrophages [25,26,27]. M1 macrophages are typically induced by interferon-gamma (IFN-γ) and microbial products such as lipopolysaccharide (LPS). They secrete pro-inflammatory cytokines like IL-12 and TNF-α, generate reactive oxygen and nitrogen species, and promote Th1 immune responses features that are generally associated with anti-tumor activity, tumor cell killing, and robust antigen presentation. In contrast, M2 macrophages arise in response to cytokines such as IL-4 and IL-10, among other factors. These cells produce anti-inflammatory cytokines like IL-10 and TGF-β, facilitate tissue remodeling and angiogenesis, and suppress adaptive immunity. Within the tumor microenvironment, TAMs frequently acquire an M2-like phenotype, thereby supporting tumor growth, metastasis, and immune evasion [28,29]. However, it is important to recognize that the M1/M2 paradigm is an oversimplification; in vivo, TAMs exhibit a broad spectrum of activation states shaped by the intricate interplay of cytokines, growth factors, hypoxia, and metabolic cues present within the TME. Recent studies have identified additional subtypes and intermediate phenotypes, underscoring the dynamic and context-dependent nature of TAM polarization [30,31,32].

TAMs contribute to cancer progression through a variety of mechanisms. They promote tumor growth and survival by secreting growth factors such as EGF, along with an array of cytokines and enzymes that enhance tumor cell proliferation [33,34]. TAMs are also central to angiogenesis, producing pro-angiogenic factors like VEGF that drive the formation of new blood vessels to nourish the tumor [35,36,37]. Additionally, TAMs facilitate metastasis by releasing matrix metalloproteinases (MMPs) and remodeling the extracellular matrix, thereby enabling tumor cell invasion and dissemination [38,39]. They exert strong immunosuppressive effects by inhibiting cytotoxic T cells and natural killer (NK) cells, producing immunosuppressive cytokines, and expressing immune checkpoint molecules such as PD-L1 [40]. Furthermore, TAMs contribute to therapy resistance by modulating drug delivery, supporting cancer stem cells, and dampening anti-tumor immune responses, collectively positioning them as critical players in tumor progression and therapeutic failure. Given their central role in cancer, TAMs have emerged as attractive targets for therapeutic intervention. Strategies under investigation include depleting TAMs with agents such as CSF-1R inhibitors, reprogramming them toward a pro-inflammatory M1-like phenotype, blocking their recruitment to the tumor site, and enhancing their phagocytic activity against tumor cells [17,18]. The innate capacity of macrophages to infiltrate solid tumors, present antigens, and modulate the immune environment provides a compelling rationale for their engineering as therapeutic agents, as exemplified by the development of CAR-macrophage (CAR-M) technology.

## 3. CAR-Macrophage Design and Engineering

**Figure 1:**
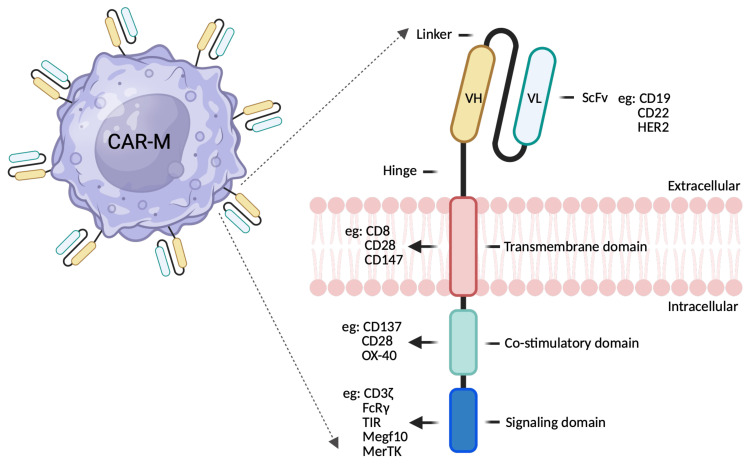
Key Structural components of CAR-Macrophages. The CAR structure in CAR-Ms consists of three core components: an extracellular domain comprising the variable regions of antibody heavy (VH) and light (VL), linked by a flexible linker forming a single-chain fragment variable (scFv) for antigen recognition and binding, an intracellular domain including both a signal transduction domain and a costimulatory domain and a transmembrane domain, and a flexible transmembrane domain plays a critical role in docking the CAR in macrophages while exposing the scFv domain for antigen binding and influencing CAT expression, stability and signal transduction. Dotted arrows indicate the blowout from the macrophage surface to the corresponding enlarged schematic of the CAR-M construct.

The successful design and engineering of CAR-macrophages (CAR-Ms) require careful consideration of several factors, including CAR construct optimization, efficient gene delivery strategies, and approaches to enhance macrophage persistence and anti-tumor activity. The CAR construct itself is a synthetic receptor that redirects immune cell specificity toward tumor-associated antigens [41,42]. While the core architecture of CARs in macrophages resembles those used in T cells, specific modifications are necessary to accommodate macrophage-specific signaling and functional requirements (Figure 1). Typically, a single-chain variable fragment (scFv) derived from an antibody is utilized to recognize tumor antigens such as HER2, CD19, or mesothelin, making the selection of an appropriate target crucial for tumor specificity and minimizing off-target effects [43,44]. The hinge and transmembrane regions, which connect the scFv to the intracellular signaling domains, are also critical, as their composition (e.g., CD8α or IgG4) influences CAR expression, stability, and function [45]. Over successive generations, CAR designs have been refined to better suit the unique demands of macrophage-based therapy (see Table 1). Unlike T cells, macrophages require activation of phagocytic and pro-inflammatory signaling pathways; thus, the intracellular domain of CARs in macrophages often incorporates motifs from phagocytic receptors (e.g., FcγR, CD3ζ, or Megf10) or costimulatory molecules such as CD28 and 4-1BB to boost activation, phagocytosis, and cytokine production [14,46]. Advanced CAR designs now include logic-gated or dual-antigen recognition systems, inducible activation modules, and domains engineered to enhance antigen presentation and T cell recruitment (see Table 2). The diversity of CAR-M designs reflects the unique biology of macrophages and the therapeutic challenges posed by solid tumors. While FcγR and Megf10 constructs remain the strongest in direct phagocytic clearance, MerTK and DAP12 offer optimal safety and balanced activation. TLR and CD3ζ-based CAR-Ms excelled in modulating the TME while synergizing effectively with T-cell immunotherapies. As gene-editing and synthetic biology approaches (e.g., IL-6 KOs, inducible circuits, dual-CAR systems) advance, CAR-Ms are poised to become versatile tools in orchestrating multi-layered anti-tumor immunity.

Achieving efficient and stable genetic engineering of macrophages remains technically challenging due to their resistance to transfection and the need to preserve their functional properties. Viral vectors, including lentiviral and adenoviral systems, are widely used for stable gene integration and high transduction efficiency, with adenoviral vectors showing promise in preclinical and early clinical studies [15]. Non-viral methods such as electroporation, mRNA transfection, and nanoparticle-based delivery offer transient gene expression and a reduced risk of insertional mutagenesis and are being optimized for improved efficiency and scalability [47,48]. Advanced gene-editing technologies like CRISPR/Cas9 enable precise knockout of inhibitory genes or introduction of modifications to enhance CAR-M function and safety [49]. Typically, peripheral blood monocytes are differentiated ex vivo into macrophages using cytokines such as M-CSF or GM-CSF before genetic modification, while induced pluripotent stem cells (iPSCs) offer a renewable and potentially standardized cell source. Establishing robust protocols for cell expansion and quality control is essential for clinical application.

Further engineering efforts focus on improving CAR-M persistence, function, and safety. This includes modifications to help CAR-Ms resist immunosuppressive signals within the TME, express supportive cytokines, or co-express survival genes. As macrophages are terminally differentiated cells with limited life span, CAR-M persistence in vivo remains as challenge. Therefore, use of CAR-monocytes, which exhibits superior circulation ability and can be differentiated into macrophages within the TME, potentially extending their therapeutic window could be a possible approach in terms of persistence [50]. Currently, this concept of using CD14-positive CAR-monocytes is being evaluated in CT-0525 Phase 1 study (NCT06254807). On a similar note, Gao et al., have shown that CAR-M derived from Hox8-inducible myeloid progenitors supports sustained in vivo production of functional CAR-M while enhancing persistence and therapeutic durability [51]. Studies on CAR-T highlighted PD-1 gene KO using CRISPR/Cas9 have showed enhanced persistence by preventing exhaustion and rapid turnover [52]. Engineering macrophages to modulate inhibitory signaling using similar strategies may provide self-renewing capabilities with the hostile TME. Their anti-tumor activity can be augmented by engineering CAR-Ms to secrete pro-inflammatory cytokines, enhance antigen presentation, or recruit endogenous T cells [50,53]. To ensure safety, incorporating suicide genes or safety switches, allowing selective depletion of CAR-Ms in the event of adverse reactions [54,55]. Manufacturing processes must be scalable and compliant with Good Manufacturing Practice (GMP) standards, with rigorous quality assurance to ensure phenotype, purity, potency, and safety of the cell product. Preclinical validation involves comprehensive in vitro assays for phagocytosis, cytokine production, antigen presentation, and T cell activation, as well as in vivo models to assess tumor infiltration, anti-tumor efficacy, and safety. Collectively, these advancements in CAR construct design, gene delivery, and functional enhancement are poised to address current challenges and unlock the full therapeutic potential of CAR-M therapies for solid tumors and beyond [56,57].

## 4. CAR-Macrophage Mechanisms of Action

**Figure 2:**
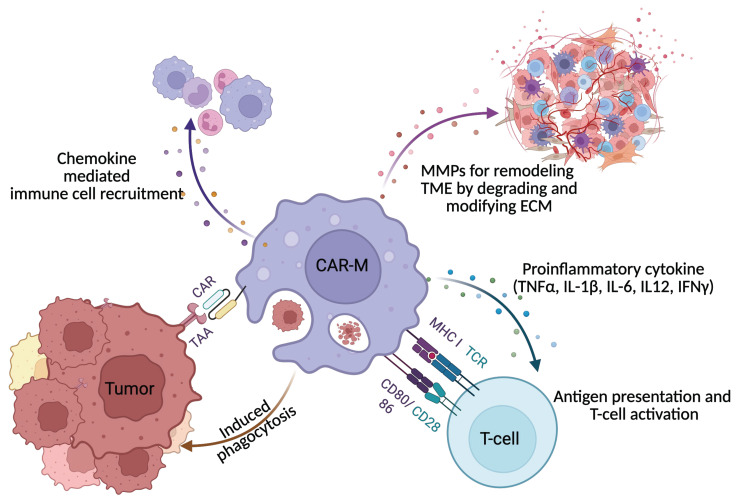
CAR-Macrophage functional mechanisms. CAR-Ms mediate antigen-specific phagocytosis through their engineered chimeric antigen receptors. They secrete pro-inflammatory cytokines to counteract the immunosuppressive tumor microenvironment and release matrix metalloproteinases (MMPs) to remodel extracellular matrix architecture. As professional antigen-presenting cells, CAR-Ms enhance T-cell activation and cytotoxicity, thereby amplifying antitumor immune responses. In addition, CAR-Ms mediate endogenous macrophages and other immune cell recruitment, further reinforcing immune activity and contributing to sustained therapeutic efficacy.

CAR-Ms are engineered to harness and redirect both the innate and adaptive immune functions of macrophages for cancer therapy (Figure 2). One of their primary mechanisms of action is direct tumor cell killing [58]. CAR-Ms are specifically designed to recognize tumor-associated antigens through their chimeric antigen receptors, which, upon antigen engagement, activate macrophages to execute potent anti-tumor effector functions. This includes enhanced phagocytosis, whereby CAR-Ms engulf and digest tumor cells expressing the target antigen, resulting in the clearance of malignant cells and the generation of tumor-derived debris that can further stimulate immune responses. Additionally, activated CAR-Ms produce cytotoxic molecules such as reactive oxygen species (ROS), nitric oxide (NO), and pro-inflammatory cytokines like TNF-α and IL-1β, all of which contribute to tumor cell lysis and apoptosis. Certain CAR-M designs also enhance antibody-dependent cellular phagocytosis (ADCP), enabling more effective elimination of antibody-opsonized tumor cells [15,59].

Beyond direct cytotoxicity, CAR-Ms play a pivotal role in remodeling the tumor microenvironment (TME), which is often highly immunosuppressive and a major barrier to effective immunotherapy. CAR-Ms secrete pro-inflammatory cytokines such as IL-12, IL-6, and TNF-α, reprogramming endogenous tumor-associated macrophages (TAMs) from a pro-tumor M2-like phenotype to an anti-tumor M1-like phenotype, thereby amplifying immune responses within the tumor [60]. They also disrupt the tumor stroma and inhibit angiogenesis by secreting matrix metalloproteinases (MMPs) and other enzymes that degrade the extracellular matrix and impair tumor vasculature, thereby limiting tumor growth and metastasis. Furthermore, CAR-Ms can neutralize immunosuppressive factors such as TGF-β, IL-10, and checkpoint ligands like PD-L1, restoring the activity of effector T cells and other immune populations. Through the release of chemokines and danger-associated molecular patterns (DAMPs), CAR-Ms also recruit and activate additional immune cells, including natural killer (NK) cells and dendritic cells, further enhancing the anti-tumor immune response [58].

A unique advantage of CAR-Ms over other CAR-engineered immune cells is their robust antigen-presenting capacity, which enables them to bridge innate and adaptive immunity (see Table 3). Following phagocytosis of tumor cells, CAR-Ms process tumor-derived antigens and present them on major histocompatibility complex (MHC) molecules to T cells—a critical function for initiating and amplifying tumor-specific T cell responses. CAR-Ms also upregulate co-stimulatory molecules such as CD80 and CD86 and secrete cytokines that promote the activation and proliferation of both cytotoxic CD8^+^ T cells and helper CD4^+^ T cells, fostering a robust systemic anti-tumor immune response. Through effective antigen presentation and T cell priming, CAR-Ms may induce long-lasting immunological memory, providing the potential for durable protection against tumor recurrence [60]. Collectively, these multifaceted mechanisms distinguish CAR-Ms from other CAR-engineered immune cells and underscore their promise, particularly in addressing the challenges posed by solid tumors characterized by immune evasion and suppression.

## 5. Preclinical and Clinical Studies

The translation of CAR-macrophage (CAR-M) therapy from concept to clinic has been propelled by a series of foundational preclinical studies and the initiation of early-phase clinical trials. Preclinical investigations have provided robust proof-of-concept for CAR-M immunotherapy, demonstrating their capacity to selectively recognize, phagocytose, and eliminate tumor cells expressing specific antigens such as HER2, CD19, or mesothelin [55,61]. In vitro studies have shown that CAR-Ms, equipped with optimized signaling domains, not only enhance phagocytic activity and pro-inflammatory cytokine production but also process and present tumor antigens to T cells, thereby bridging innate and adaptive immunity [53,62,63]. Similarly, in vivo studies have exhibited efficient infiltration into solid tumors, resulting in significant tumor burden reduction and prolonged survival in mouse models of breast, ovarian, and pancreatic cancer [64,65,66]. These studies further highlight the ability of CAR-Ms to remodel the tumor microenvironment (TME) by reprogramming endogenous macrophages toward a pro-inflammatory state, reducing immunosuppressive factors, and increasing T cell infiltration. Importantly, safety profiles in animal models have generally been favorable, with minimal off-target toxicity. Notably, the study by Klichinsky et al., employing HER2-specific CAR-Ms, set a benchmark for the field, demonstrating both potent anti-tumor activity and effective TME modulation [53].

Building on these preclinical successes, early-phase clinical trials are now evaluating the safety, feasibility, and preliminary efficacy of CAR-M therapies in humans (see Table 4). The most advanced trial to date is CT-0508, a phase 1 study sponsored by Carisma Therapeutics, testing autologous HER2-targeted CAR-Ms in patients with advanced HER2-positive solid tumors. Initial results indicate that CT-0508 is well tolerated, with manageable side effects, no dose-limiting toxicities observed in early cohorts, and evidence of immune activation within the TME [46]. Additional clinical programs are ongoing, targeting other antigens, exploring allogeneic or iPSC-derived CAR-Ms, and investigating combination regimens with checkpoint inhibitors and other immunotherapies. Despite these advances, challenges such as manufacturing scalability, in vivo persistence, and optimal dosing remain key considerations for the continued clinical development of CAR-M therapies.

## 6. Preclinical Promise Versus Clinical Uncertainty in CAR-Macrophage Therapy

Motivated by the limitations of lymphocyte-based therapies in solid tumors, CAR-M strategies leverage the natural abundance of macrophages within tumors, where they readily infiltrate hypoxic and stromal dense regions and possess intrinsic phagocytic and antigen-presenting capabilities. Complementing this rationale, preclinical studies have also demonstrated that CAR-Ms can be genetically programmed to recognize tumor-associated antigens, resulting in enhanced phagocytosis, secretion of pro-inflammatory cytokines, and secondary activation of adaptive immune responses through efficient antigen cross-presentation [53]

Despite the strong preclinical rationale, the clinical translation of CAR-M therapies faces several technical and biological challenges. Although the first-in-human CAR-M programs (e.g., CT0508) reported manageable safety profiles, with notably minimal CRS as compared to CAR-T therapy, objective clinical responses have been modest, with most studies reporting stable disease rather than durable tumor regressions [46]. Additionally, macrophages exhibit limited persistence in vivo due to their restricted proliferative capacity after transfer, which may compromise long-term efficacy [48]. Thus, while feasibility and safety have been demonstrated, clinical efficacy remains uncertain, and the gap between animal models and patients has become increasingly apparent.

A central barrier to CAR-M translation is the intrinsic plasticity of macrophages [15]. Unlike T cells, macrophages continuously integrate local environmental cues, raising substantial concerns about phenotype stability after infusion. Pro-inflammatory CAR-Ms generated in vitro may be rapidly reprogrammed by the TME into immunosuppressive states, eroding engineered functionality. Preclinical models inadequately capture the multifactorial pressures of human tumors, where chronic hypoxia, metabolic stress, lactic acid accumulation, and immunosuppressive cytokines such as TGF-β and IL-10 impose sustained conditioning of myeloid cells. This environmentally enforced plasticity introduces translational uncertainty that is far less pronounced in CAR T cell therapy [67,68].

Manufacturing challenges further undermine consistency and scalability [15]. The production process includes monocyte isolation, differentiating them into macrophages, genetic modification, and expansion under stringent GMP conditions, each of which introduces potential variability and inconsistency. Macrophages are terminally differentiated, non-proliferative, and highly sensitive to ex vivo manipulation, resulting in comparatively low viral transduction efficiencies [9,18]. Dependence on alternative delivery approaches, including adenoviral or non-viral vectors, raises unresolved concerns regarding transgene durability and immunogenicity. Additionally, significant donor-to-donor variability in monocyte starting material introduces batch heterogeneity, complicating GMP standardization and reproducibility, the key determinants of the clinical success of CAR T manufacturing platforms.

Biologically, CAR macrophages also differ fundamentally from CAR T cells in their mechanisms of action, relying primarily on phagocytosis, cytokine-mediated effects, and immune orchestration rather than rapid cytolysis. As a result, translating preclinical tumor control into clinically meaningful RECIST responses may require reconsideration of endpoints, dosing strategies, or combination regimens [50,69]. Antigen selection remains problematic, as few tumor antigens can drive effective phagocytosis without provoking damaging off-tumor inflammation, an especially acute concern given macrophages’ potent inflammatory capacity. The divergence between preclinical efficacy and clinical uncertainty in CAR-M therapy reinforces a fundamental lesson in immuno-oncology that biological plausibility does not necessarily translate into clinical success. Consequently, while CAR-M therapies offer compelling therapeutic advantages, overcoming technical, biological, and manufacturing hurdles is essential to fully realize their transformative potential in cancer treatment.

## 7. Emerging Strategies and Innovations

As CAR-M therapy advances toward clinical application, researchers are developing innovative engineering strategies to overcome current limitations and enhance therapeutic efficacy, particularly for solid tumors. At the forefront of these efforts are novel CAR designs, including dual-targeting CARs capable of recognizing two distinct tumor antigens, thereby increasing specificity and reducing the risk of tumor escape. Some optimized CAR-M constructs combine multiple mechanisms, such as coupling a phagocytosis-inducing CAR with an auto-secreted CD47 blocker to counteract the “don’t eat me” signals exploited by tumors, while also facilitating cross-priming of endogenous T cells to generate a secondary wave of immune attack against heterogeneous tumor clones [10,70]. Logic-gated CARs, which utilize Boolean logic gates (AND, OR, NOT), allow for highly specific activation only under defined conditions, enhancing both safety and precision by requiring co-expression of multiple tumor antigens or inhibiting activation in healthy tissues. Inducible and tunable CARs, responsive to external cues such as small molecules, offer clinicians greater control over CAR-M activity. In parallel, next-generation CARs incorporate enhanced intracellular signaling domains from innate immune receptors or costimulatory molecules, further optimizing macrophage activation, cytokine production, and antigen presentation.

Beyond advanced CAR constructs, combination therapies are being actively explored to maximize the anti-tumor potential of CAR-Ms. Immune checkpoint inhibitors, such as anti-PD-1 or anti-CTLA-4 antibodies, can relieve immunosuppression within the TME and synergize with CAR-Ms to boost endogenous T cell responses [55,71]. Oncolytic viruses, which selectively infect and lyse tumor cells, can enhance antigen release and immune cell infiltration, further potentiating CAR-M efficacy [72]. Conventional therapies like chemotherapy and radiotherapy may also be combined with CAR-Ms to modulate the TME, upregulate target antigens, and induce immunogenic cell death. Additionally, combinatorial strategies with other cellular therapies including CAR-T and CAR-NK cells are being investigated to harness the unique strengths of each cell type in a coordinated anti-tumor response [55,64,73].

Synthetic biology and next-generation engineering approaches are expanding the functional repertoire of CAR-M therapy. Armored CAR-Ms are engineered to secrete immunostimulatory cytokines, chemokines, or antibodies to recruit and activate endogenous immune cells or counteract immunosuppressive signals within the TME. Advanced gene editing tools such as CRISPR/Cas9 enable precise modifications to enhance CAR-M persistence, activity, and safety, including the knockout of inhibitory genes or conferring resistance to suppressive TME factors. Universal and off-the-shelf CAR-M products derived from allogeneic, or iPSC sources are being developed to improve scalability and accessibility, with additional modifications to evade immune rejection. Integration of biosensors and genetic circuits allows CAR-Ms to sense environmental cues and deliver tailored therapeutic responses, while enhanced antigen presentation machinery further stimulates adaptive immunity and promotes durable anti-tumor responses [74]. Collectively, these emerging strategies are rapidly advancing the therapeutic landscape of CAR-M therapy, promising safer, more effective, and widely applicable treatments for cancer.

## 8. Future Perspectives

CAR-M immunotherapy stands at the forefront of cancer treatment innovation, offering renewed hope for malignancies that have proven resistant to both conventional and advanced immunotherapies. Its most promising clinical applications are in solid tumors such as breast, ovarian, pancreatic, and lung cancers, as well as glioblastoma and melanoma where the innate ability of CAR-Ms to infiltrate and persist within the tumor microenvironment provides a distinct therapeutic advantage [15]. Beyond solid tumors, CAR-Ms may also play a role in hematological malignancies, particularly in settings where antigen presentation and modulation of the tumor microenvironment are critical [75]. Their capacity to reshape the immune landscape and activate adaptive immunity positions CAR-Ms as candidates for treating metastatic and recurrent disease, and for use in combination or adjunctive regimens with checkpoint inhibitors, oncolytic viruses, chemotherapy, or other cellular therapies to maximize therapeutic benefit.

As CAR-M therapies advance toward broader clinical application, regulatory and ethical considerations become increasingly important. Ensuring patient safety requires comprehensive risk assessments, including evaluation of off-target effects, immunogenicity, cytokine profiles, and long-term persistence. Manufacturing processes must adhere to rigorous GMP standards [76] to ensure product consistency and quality, while robust informed consent procedures are essential to help patients understand the risks and benefits associated with these novel therapies. The complexity and cost of personalized cell therapies also raise concerns about equitable access, underscoring the need for scalable, off-the-shelf CAR-M products to minimize disparities in care. Additionally, the use of advanced gene-editing technologies prompts important ethical questions regarding the extent of genetic modification and the safeguarding of patient genetic information.

Looking ahead, several key research priorities will shape the future of CAR-M immunotherapy. These include the identification and validation of novel tumor-specific antigens to enhance efficacy and safety, the optimization of CAR-M engineering and gene delivery techniques, and a deeper understanding of how CAR-Ms interact with the immune system and the tumor microenvironment. Overcoming immunosuppressive barriers within tumors, assessing long-term outcomes and the potential for immunological memory, and expanding applications beyond oncology such as in infectious diseases or autoimmune disorders are also critical areas for ongoing investigation. Ultimately, the future of CAR-M therapy is highly promising, with the potential to revolutionize cancer care and extend the benefits of cellular immunotherapy to a broader patient population, provided that continued innovation, rigorous evaluation, and ethical oversight remain central to its development.

## 9. Conclusions

CAR-Macrophage (CAR-M)-based immunotherapy represents a significant and innovative advancement in cancer treatment, particularly for solid tumors where existing immunotherapies often fall short. This review has highlighted the unique biological attributes of macrophages including their innate capacity for tumor infiltration, robust phagocytic activity, and ability to modulate the tumor microenvironment and activate adaptive immunity. These features set CAR-Ms apart from other CAR-engineered immune cells and position them as promising candidates for overcoming the formidable barriers associated with solid malignancies. Compelling evidence from preclinical studies supports the efficacy of CAR-Ms, while early-phase clinical trials, though still in their initial stages, suggest that CAR-M therapies are both feasible and exhibit manageable safety profiles. Ongoing and future studies are expected to provide critical insights into their therapeutic potential and long-term outcomes.

Despite this progress, several challenges remain before CAR-M therapies can achieve widespread clinical adoption. Technical hurdles such as efficient genetic engineering, reliable cell sourcing, and scalable manufacturing processes are significant obstacles. Biologically, ensuring in vivo persistence, minimizing off-target effects, and overcoming the immunosuppressive signals within the tumor microenvironment require continued innovation and rigorous investigation. Additionally, regulatory and ethical considerations, particularly those related to safety, equitable access, and the implications of genetic modification, must be thoughtfully addressed. Looking forward, the integration of novel CAR designs, combination strategies, and synthetic biology approaches holds considerable promise for enhancing the efficacy, safety, and accessibility of CAR-M immunotherapy. Continued research into CAR-M mechanisms of action, the optimization of engineering and manufacturing workflows, and well-designed clinical trials will be essential to fully realize the transformative potential of this emerging therapeutic modality.

## Figures and Tables

**Table 1:** Generations of CAR-Macrophage design in immunotherapy.

Generation	CAR Design Focus	Key Strengths	Limitations
First	T cell-style CARs (CD3ζ, ±CD28/4-1BB)	Enhanced antigen presentation; Activates macrophage APC function; High safety profile	Weak phagocytosis; Not optimized for myeloid signaling
Second	Phagocytic receptor CARs (e.g., FCγR, Megf10, MERTK, DAP12)	Strong engulfment; Balanced activation; Low inflammation	Some constructs produce local inflammatory cytokines; Requires antigen accessibility for phagocytosis
Third	TME reprogramming CAR-M (e.g., CD40L, TLR, Cytokine secreting)	Potent TMR modeling; Drives M1 like polarization; Recruits and primes T cells	Higher inflammatory potential; Required tuned regulation for safety
Fourth	Gene edited armored CAR-M (e.g., IL-6 KO, PD-1 DNR, SIRPα KO)	Low CRS risk; Enhanced persistence; Improved specificity	Increased manufacturing complexity; Needs validated safety switches
Fifth	Synthetic circuit CAR-M (Logic gated, inducible, multi-input designs)	Highly specific tumor targeting; Minimal-off tumor activation;	Preclinical stage; Complex validation and safety validation

**Table 2:** Comparative overview of CAR-Macrophage design.

CAR Design	Strength	Limitation	Therapeutic Application
FcγR-based (CD32A/CD64 ITAMs)	Strong phagocytosis; Robust Syk-mediated activation; Effective in antibody resistant tumors	Higher local inflammatory cytokines (TNFα, IL-1β); Requires careful control in inflamed tissue	Solid tumors with high antigen density; Tumors requiring potent direct clearance
Megf10	Efficient Efferocytosis; Minimal inflammatory cytokine release; High safety and tissue compatibility	Limited TME remodeling compared to TLR-based and CD40L-enhanced CAR-M	Tumors near sensitive tissues (CNS, GI tract); Situations requiring strong but quite phagocytosis
MerTK	Moderate phagocytosis with very low inflammation; Promotes M1-like reprogramming under engineered conditions; Good balance of safety and efficacy	Less aggressive tumor clearance than FcγR or Megf10; May require co-therapies to maximize TME remodeling	Tumors with immunosuppressive TME; combination strategies with checkpoint inhibitor CAR-T
TLR (TLR2/4 fusion)	Potent M1 polarization; High secretion of IL-2, TNFα, and ROS, Strong TME remodeling	High inflammatory potential, Requires safeguard mechanisms for safety	Immunologically cold tumors, Tumors needing immune activation and T-cell recruitment
CD3ζ (T-cell)	Enhanced antigen presentation and cross priming; low toxicity; Synergizes with adaptive immunity	Weak phagocytosis; limited autonomous tumor killing	Combination with CAR-T, vaccines, checkpoint therapy; TME activation
DAP12	Balanced ITAM signaling; Moderate-strong phagocytosis; Lower inflammatory footprint than FcγR	Less potent antigen uptake than FcγR; Moderate cytokine induction	Solid tumors needing controlled activation; Myeloid-enrichment tumors
CD40L	Superior T-cell recruitment through CD40-CD40L axis; Strong APC function; Combats suppressive TMEs	Requires tight regulation due to APC activation; Unwanted local inflammation	Solid tumors requiring T-cell priming and recruitment; improving immunotherapy response rates
IL-6 KO	Enhanced safety via reduced CRS cytokines; Suitable for systemic delivery; Improved persistence	Requires genome editing; Complex manufacturing	High-risk patients; multi-dose strategies; Solid tumors requiring sustained macrophage activity
Next-generation synthetic Circuit CAR-M (logic-gated, inducible)	Tumor specific activation (AND/NOT gates); High precision; programmable TME reshaping	Still preclinical; Needed complex engineering and validation	Precision solid tumor targeting; Deep-tissue tumors; Scenarios requiring spatial or temporal control

**Table 3:** Comparison highlighting the key features, advantages and challenges of CAR-T, CAR-NK and CAR-Macrophages.

Features	CAR-T	CAR-NK	CAR-M
Origin	Autologous or allogeneic T lymphocytes	Autologous, allogeneic, or cell lines (e.g., NK-92)	Monocyte-derived or cell line macrophages
Primary Mechanism	Cytokine killing via perforin/granzyme, cytokine release	Cytokine killing via perforin/granzyme, ADCC, cytokine release	Phagocytosis, cytokine release, antigen presentation
Tumor Infiltration	Moderate	Moderate to good	Excellent
Antigen Presentation	Limited	Limited	Strong
Cytokine Release Syndrome (CRS) Risk	High	Lower than CAR-T	Very low
Graft-vs-Host Disease (GvHD) Risk	Possible	Very low	Very low
	Limited (mainly autologous)	High (allogeneic, cell lines feasible)	Moderate (potential for allogeneic products)
Current Clinical Status	FDA-approved for several cancers	Early-phase clinical trails	Preclinical and early-phase clinical trails
Main Advantages	Proven efficiency in hematologic cancers, long-term persistence	Lower CRS risk, off-the-shelf potential, innate immunity	TME modulation, phagocytosis, antigen presentation
Main Challenges	CRS, neurotoxicity, limited efficiency in solid tumors	Persistence, expansion, transduction efficiency	Optimization of CAR design, in vivo persistence and safety

**Table 4:** Clinical trials on CAR-macrophages (CAR-Ms).

Trial Name/Identifier	Sponsor	Indication	Target Antigen	CAR-M Source	Phase	Status
CT-1119 NCT0572596	Carisma Therapeutics, Philadelphia, PA, USA	Mesothelin + Solid Tumors primarily focusing on patients with ovarian and pancreatic cancers	Mesothelin	Autologous	Phase 1	Actively advancing in late 2025
NCT05138458	CREATE Medicines, Inc., Cambridge, MA, USA, formerly known as Myeloid Therapeutics, Inc.	CD5+ Advanced solid tumors	CD5	Autologous	Phase 1/2	Active
NCT06224738	NCI, NIH grant P30DK056338	HER2+ Solid Tumors	Advanced HER2+ gastric cancer and peritoneal metastases	Autologous	Phase 1	Active
CT-0525 NCT06254807	Carisma Therapeutics	HER2+ Solid Tumors	HER2	Autologous	Phase 1	Active
ChiCTR2400082776/ChiCTR2400080078	First People’s Hospital of Hangzhou	HER2-positive and HER2-low solid tumors.	HER2	Autologous	Phase 1	Active
MCY-M11 NCT03608618	MaxCyte, Inc. Rockville, MD, USA/CARMA Cell Therapies	Mesothelin expressing solid ovarian and peritoneal mesothelioma	Mesothelin	Autologous	Phase 1; dose-escalation study	Completed
CT-0508 NCT04660929	Carisma Therapeutics	HER2+ Solid Tumors	HER2	Autologous	Phase 1	Completed

## Data Availability

No new data were created or analyzed in this study. Data sharing is not applicable to this article.
